# Viral Metagenomic Analysis of Cerebrospinal Fluid from Patients with Acute Central Nervous System Infections of Unknown Origin, Vietnam

**DOI:** 10.3201/eid2701.202723

**Published:** 2021-01

**Authors:** Nguyen To Anh, Le Nguyen Truc Nhu, Nguyen Thi Thu Hong, Tran My Phuc, Pham Thi Thanh Tam, Dang Thao Huong, Tran Tuan Anh, Xutao Deng, Ho Dang Trung Nghia, Tran Thua Nguyen, Nguyen Van Hung, Nguyen Dac Thuan, Pham Thi Hong Phuong, Nguyen Van Vinh Chau, Stephen Baker, Eric Delwart, Guy Thwaites, Le Van Tan, for the VIZIONS Consortium

**Affiliations:** Oxford University Clinical Research Unit, Ho Chi Minh City, Vietnam (N.T. Anh, L.N.T. Nhu, N.T.T. Hong, T.M. Phuc, P.T.T. Tam, D.T. Huong, T.T. Anh, H.D.T. Nghia, G. Thwaites, L.V. Tan);; Hue Central Hospital, Hue City, Vietnam (T.T. Nguyen);; Dak Lak General Hospital, Ban Me Thuot City, Vietnam (N.V. Hung);; Khanh Hoa General Hospital, Nha Trang City, Vietnam (N.D. Thuan);; Dong Thap General Hospital, Dong Thap, Vietnam (P.T.H. Phuong);; Vitalant Research Institute, San Francisco, California, USA (X. Deng, E. Delwart);; University of California Department of Laboratory Medicine, San Francisco (X. Deng, E. Delwart);; Pham Ngoc Thach University, Ho Chi Minh City (H.D.T. Nghia);; Hospital for Tropical Diseases, Ho Chi Minh City (N.V.V. Chau);; University of Cambridge Institute of Therapeutic Immunology and Infectious Disease, Cambridge, UK (S. Baker);; Centre for Tropical Medicine, Nuffield Department of Medicine, University of Oxford, Oxford, UK (S. Baker, G. Thwaites)

**Keywords:** central nervous system infection, viral metagenomics, enterovirus, Vietnam, next-generation sequencing, viruses, meningitis/encephalitis

## Abstract

Central nervous system (CNS) infection is a serious neurologic condition, although the etiology remains unknown in >50% of patients. We used metagenomic next-generation sequencing to detect viruses in 204 cerebrospinal fluid (CSF) samples from patients with acute CNS infection who were enrolled from Vietnam hospitals during 2012–2016. We detected 8 viral species in 107/204 (52.4%) of CSF samples. After virus-specific PCR confirmation, the detection rate was lowered to 30/204 (14.7%). Enteroviruses were the most common viruses detected (n = 23), followed by hepatitis B virus (3), HIV (2), molluscum contagiosum virus (1), and gemycircularvirus (1). Analysis of enterovirus sequences revealed the predominance of echovirus 30 (9). Phylogenetically, the echovirus 30 strains belonged to genogroup V and VIIb. Our results expanded knowledge about the clinical burden of enterovirus in Vietnam and underscore the challenges of identifying a plausible viral pathogen in CSF of patients with CNS infections.

Worldwide, the annual incidence of acute encephalitis in nonoutbreak settings during 1983–2000 ranged from 0.07 to 12.6 cases/100,000 population (*1*). According to the World Health Organization, meningitis caused 379,000 deaths and encephalitis caused 150,000 deaths globally in 2015 (*2*). As a consequence, central nervous system (CNS) infection is a leading cause of years lived with disability in low-income countries (*3*).

More than 100 known pathogens can cause CNS infections (*1*). However, the distribution of CNS infection pathogens is geographically dependent and has been shaped by the emergence of novel viruses. In Asia, Nipah virus and enterovirus A71 have been recognized as emerging neurotropic pathogens over the past few decades. In 1999, West Nile virus arrived in the United States and since then has established endemic circulation (*4*).

Despite recent advances in molecular diagnostics, especially sensitive virus-specific PCR, encephalitis cases of unknown origin remain a substantial problem. Worldwide, ≈50% of patients with CNS infections have no etiology identified (*1*,*5*,*6*).

Over the past decade, metagenomic next-generation sequencing (mNGS) has emerged as a sensitive hypothesis-free approach for detection of pathogens (especially viruses) in clinical samples (*7*). However, in resource-limited settings like Southeast Asia and Vietnam, a limited number of mNGS studies examining known and unknown viruses in cerebrospinal fluid (CSF) samples from patients with CNS infections have been conducted, even though in this tropical region of the world, novel viruses are likely to emerge (P. Zhou et al., unpub. data, https://doi.org/10.1101/2020.01.22.914952), and diverse CNS infection pathogens have been documented. Collectively, improving our knowledge about viral causes of CNS infections is essential for clinical management and development of intervention strategies. In this study, by using a mNGS approach, we set out to search for known and unknown viruses in CSF samples collected from patients in Vietnam with CNS infections of unknown causes who were enrolled in a hospital-based surveillance study conducted during 2012–2016.

## Materials and Methods

### Clinical Study and Selection of CSF Samples for mNGS Analysis

The study used CSF samples collected from patients with suspected CNS infection enrolled in a hospital-based surveillance program conducted in Vietnam during December 2012–October 2016 (*5*). The study was conducted as part of the Vietnam Initiative on Zoonotic Infections (VIZIONS) project (*5*), and patient recruitment was carried out at 7 provincial hospitals across Vietnam. After collection, as per the study protocol, all CSF samples were tested for a range of pathogens by using the diagnostic work-up of the clinical study (Appendix Table 1). The remaining volume of the CSF samples were stored at −80°C for further testing.

**Figure 1:**
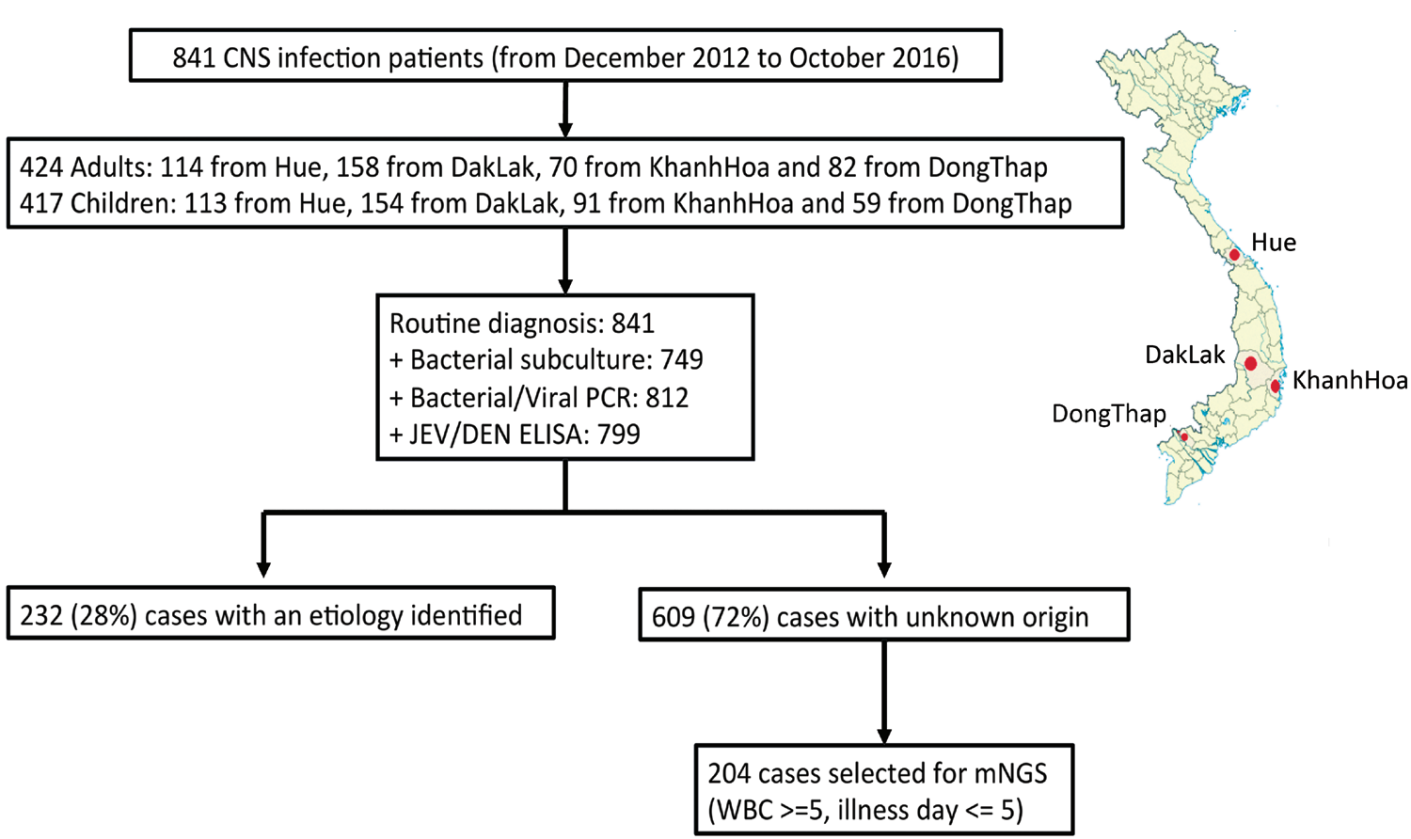
Flowchart overview of diagnostic results for study of patients with suspected central nervous system infections admitted to 4 of 7 provincial hospitals, Vietnam, December 2012–October 2016. Inset map indicates places where samples were collected (red dots).

We focused our metagenomic analysis on patients of unknown origin from 4 provincial hospitals in central (Hue and KhanhHoa), highland (DakLak), and southern (DongThap) Vietnam (Figure 1), representing 3 distinct geographic areas in Vietnam. To increase the chance of detecting a virus in the CSF samples, we only selected patients with CSF leukocyte counts >5 cells/mm^3^ and an illness duration <5 days.

### mNGS Assay

mNGS assay was carried out as previously described (*8*). Before viral nucleic acid (NA) isolation, 100 μL of each CSF sample was treated with Turbo DNase (Ambion, Life Technology, ThermoFisher, https://www.thermofisher.com) and RNase I enzyme (Ambion). Then viral NA was isolated using a QIAamp viral RNA kit (QIAGEN GmbH, https://www.qiagen.com), and recovered in 50 μL of elution buffer provided with the extraction kit. Double-stranded DNA was synthesized from the isolated viral NA by using a set of 96 nonribosomal primers (FR26RV–Endoh primers) and then was randomly amplified by using the FR20RV primer (5′-GCCGGAGCTCTGCAGATATC-3′). Finally, the amplified product was subjected to a library preparation step by using Nextera XT sample preparation kit (Illumina, https://www.illumina.com), following the manufacturer’s instructions, and sequenced by using a MiSeq reagent kit, version 3 (600 cycles) (Illumina) in a MiSeq platform (Illumina).

### mNGS Data Analysis

Potential viral reads were identified by using an in-house viral metagenomic pipeline running on a 36-node Linux cluster as described previously (*9*). In brief, after duplicate reads and reads belonging to human or bacterial genomes were filtered out, the remaining reads were assembled de novo. The resulting contigs and singlet reads were then aligned against a customized viral proteome database by using an approach based on BLAST (https://blast.ncbi.nlm.nih.gov/Blast.cgi). Next, the candidate viral reads were aligned against a nonredundant nonvirus protein database to remove any false-positive reads (i.e., reads with expected values higher than those against viral protein databases). Any virus-like sequence with an expected value <0.00001 was considered a significant hit. Finally, a reference-based mapping approach (Genious 8.1.5; Biomatters, https://www.geneious.com) was used to assess the levels of identity and genome coverage of the corresponding viruses.

### PCR Confirmatory Testing of mNGS Results

PCR assays were conducted to confirm mNGS hits for each specific virus identified from the viral metagenomic pipeline. Depending on availability of CSF, the PCR confirmations were performed either on leftover NA or newly extracted NA. A viral mNGS result was considered positive only if it was subsequently confirmed by PCR analysis of the original NA samples. The nucleotide sequences of primers and probes used for PCR confirmatory testing are shown in Appendix Table 2 (*8*).

### Serotype Identification and Phylogenetic Analysis

For enterovirus serotype determination based on the obtained sequences generated by viral mNGS, we used a publicly available genotyping tool (*10*). To determine the relationship between enterovirus strains we sequenced and global strains, we first performed pairwise alignment by using the ClustalW tool in Geneious 8.1.5, and then reconstructed a maximum-likelihood phylogenetic tree by using IQ Tree 1.4.3 (*11*). A similar phylogenetic approach was used for other viruses. The generated sequences of this study were submitted to GenBank (accession no. PRJNA561465).

### Ethics

The study was approved by the corresponding institutional review broad of local hospitals in Vietnam, where the patients were enrolled, and the Oxford Tropical Ethics Committee. Informed consent was obtained from each study participant or a legal guardian.

## Results

### CSF Samples Available for mNGS Analysis

From the clinical study described previously, a total of 841 patients were enrolled from Hue, Khanh Hoa, Dak Lak, or Dong Thap provincial hospitals. Of these, 609/841 (72%) patients had no etiology identified. The etiologic profiles of the patients in whom a pathogen was detected will be reported separately. Of the patients in whom a pathogen was not identified, 204 met our selection criteria, and their CSF samples were included for viral mNGS analysis (Figure 1).

### Baseline Characteristics of the Included Patients

The baseline characteristics and outcome of the 204 study patients are described in Table. Male patients were predominant. A substantial proportion of the patients were seriously ill; fatal outcome was recorded in 22 (11%), whereas incomplete recovery was recorded in 17% (n = 35) and deterioration (reflected by being transferred to other hospitals) in 16.5% (n = 34).

**Table:** Baseline characteristics and clinical data of patients with acute central nervous system infections enrolled for mNGS analysis of CSF samples, Vietnam, December 2012–October 2016*

Characteristic	Patients with unknown cause enrolled for mNGS, n = 204	Patients with mNGS negative, n = 174	Patients with enterovirus detected, n = 23	p value†
Sex				
M	135 (66)	114 (65.5)	15 (65)	
F	69 (34)	60 (34.5)	8 (35)	
Age, y, median (range)	20.5 (0–92)	24 (0–92)	13 (2–27)	0.005
Location				
Hue	37 (18)	28 (16)	9 (39)	
Dak lak	98 (48)	87 (50)	10 (43.5)	
Khanh Hoa	28 (14)	22 (13)	4 (17.5)	
Dong Thap	41 (20)	37 (21)	0	
3-d fever (at enrollment or preceding 3 d)				
Fever	148 (72.5)	126 (72.4)	17 (74)	0.054
Temperature, C°, median (range)	39 (37.5–42.0)	39 (37.5–42.0)	38.5 (38.0–40.5)	
Fever with unknown temperature	29 (14.2)	22 (12.6)	6 (26)	
No fever	20 (9.8)	19 (11)	0	
Unknown	7 (3.5)	7 (4)	0	
Outcome				
Death or discharge to die	22 (11)	22 (12.6)	0	
Discharge with complete recovery	108 (53)	86 (49.4)	18 (78.3)	
Discharge with incomplete recovery	35 (17)	31 (17.8)	2 (8.7)	
Transfer to another hospital	34 (16.5)	30 (17.2)	3 (13)	
Other (patient request)	3 (1.5)	3 (1.7)	0	
Unknown	2 (1)	2 (1.3)	0	
CSF white cells, cells/mm^3^ median (min–max)	88.5 (5–40,000)	71.5 (5–40,000)	110 (8–1200)	0.343

*Values are no. (%) except as indicated, CSF, cerebrospinal fluid; mNGS, metagenomic next-generation sequencing. †Statistical comparisons were performed for groups of patients with mNGS-negative results and enterovirus detected, by Mann-Whiney test.

### General Description of mNGS Results

**Figure 2:**
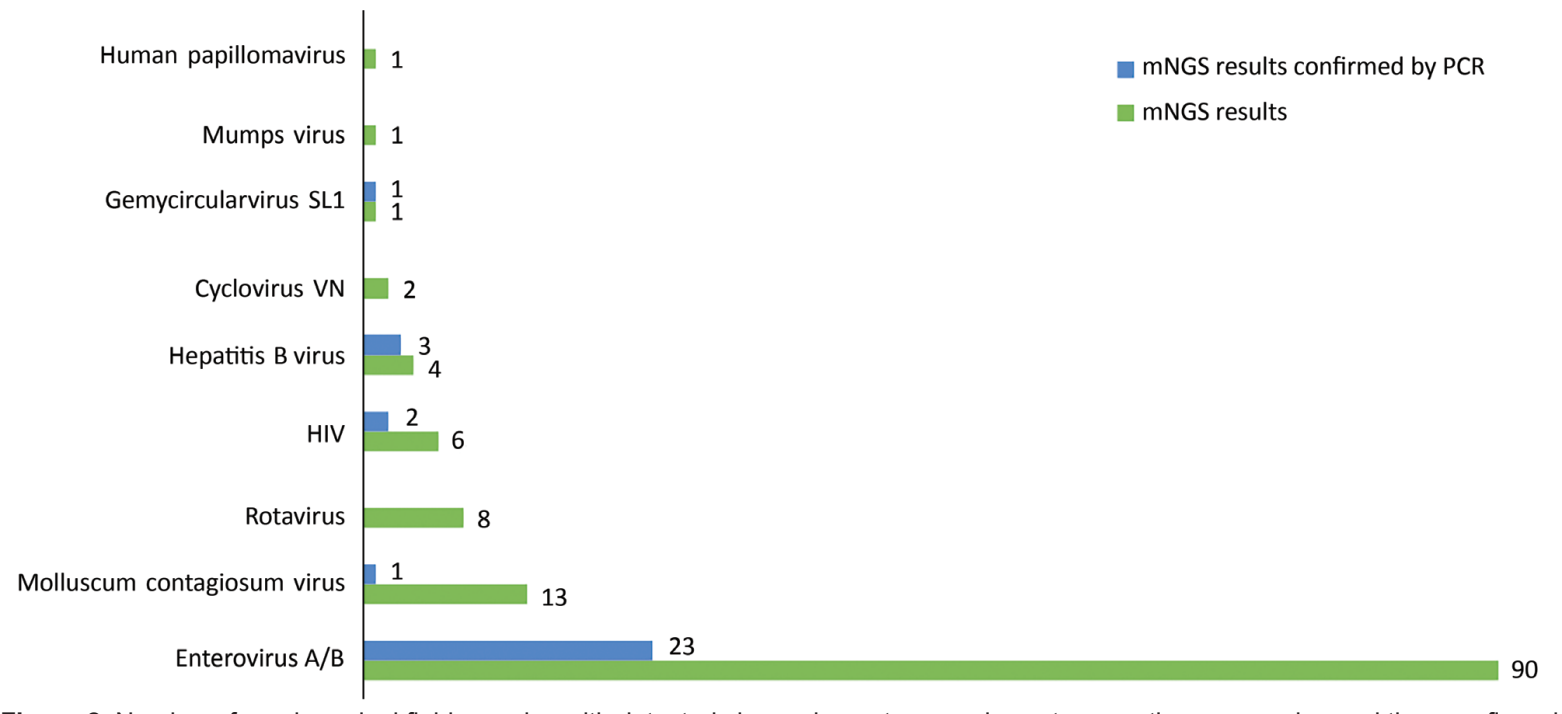
Number of cerebrospinal fluid samples with detected viruses by metagenomic next-generation sequencing and then confirmed by virus-specific PCR or reverse-transcription PCR, Vietnam, December 2012–October 2016. Samples were collected from patients with suspected central nervous system infection. For human papillomavirus, confirmatory testing was not performed because of the unavailability of a PCR assay.

A total of 204 CSF samples were subjected to 3 NGS runs, and 108 million reads were obtained (median number of reads per sample 445,412 [range 430–908,890]). Of these, viral reads accounted for 0.64% (n = 692,731; median number of reads per sample 2,001 [range 4–268,933]). Excluding common contaminants and commensal viruses such as torque teno virus, which are not reported in this article, sequences related to a total of 8 distinct viral species were identified in 107/204 (52.4%) patients. These viruses are either known to be infectious to humans (e.g., enteroviruses, rotavirus, molluscum contagiosum virus [MCV], human papillomavirus, HIV, and hepatitis B virus [HBV]) or are without evidence of human infections besides previous detection in sterile human samples (e.g., cyclovirus-VN and gemycircularvirus) (Figure 2).

### mNGS Result Assessment by Specific PCR Analysis

After virus-specific PCR confirmatory testing, the proportion of patients in whom a virus was found by mNGS was reduced from 53% (108/204) to 14.7% (30/204). Accordingly, the number of virus species was reduced from 8 to 5 (Figure 2); enteroviruses were the most common virus detected, accounting for 11.3% (23/204) of the included patients, followed by HBV (n = 3), HIV (n = 2), gemycircularvirus, and MCV (1 each) (Figure 2). Because of the focus of our study and the unavailability of the PCR assays, confirmatory testing for human papillomavirus was not performed.

### Characteristics of the 23 Enterovirus-Infected Patients

All 23 enterovirus-infected patients were admitted to hospitals from the central or highland areas (Table), and none were from Dong Thap Province. Male patients were slightly predominant, accounting for 56%. Notably, the enterovirus-infected patients were younger than those who were mNGS-negative (Table). At discharge, incomplete recovery or transfer to other hospitals because of disease deterioration were recorded in 21.7% (Table).

**Figure 3:**
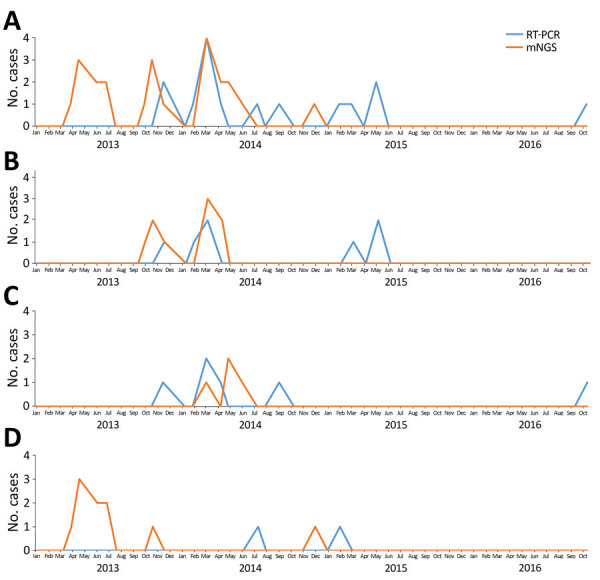
Temporal distribution of enterovirus cases detected from cerebrospinal fluid samples of patients with suspected central nervous system infection by metagenomic next-generation sequencing and RT-PCR, Vietnam, December 2012–October 2016. Enterovirus RT-PCR results were obtained from the original study. RT-PCR, reverse transcription PCR. A) Combined data from 3 provinces; B) data from Hue province; C) data from Khanh Hoa province; D) data from Dak Lak province.

Enterovirus cases were not detected during January 2015–December 2016. During 2013 and 2014, two main peaks were observed during March–July and September–December (Figure 3, panel A); cases from Dak Lak and Khanh Hoa contributed to the first peak (Figure 3, panels B and C), and cases from Khanh Hoa and Hue contributed to the second (Figure 3, panels C and D). The general baseline characteristics of patients with HBV, gemycircularvirus, and MCV are shown in Appendix Table 3.

### Genetic Characterization of Enteroviruses and Gemycircularvirus

**Figure 4:**
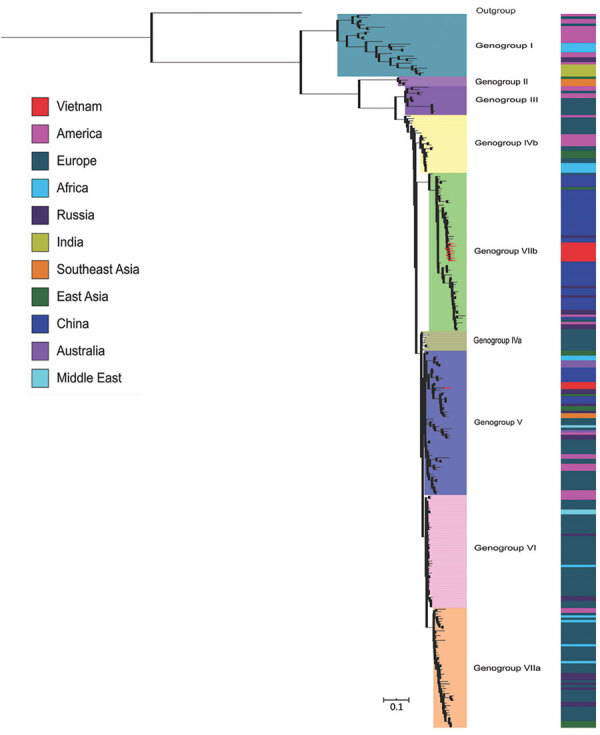
Phylogenetic tree of 298 complete viral protein 1 sequences of echovirus 30 (876 nt) isolated from cerebrospinal fluid samples of patients with suspected central nervous system infection, Vietnam, December 2012–October 2016. The inner color strip indicates 7 genogroups. The outer colorstrip indicates different countries of echovirus 30 isolates included in the tree. The outgroup is echovirus 21 Farina.

mNGS generated sufficient sequence information for an enterovirus serotyping assessment in 11/23 cases. Subsequently, results of serotyping analysis based on the NGS sequences showed that echovirus 30 (E30) was the most common serotype detected (n = 9, 39% of enteroviruses), followed by enterovirus A71 and enterovirus B80 (1 each, 4.3%). Phylogenetically, the 9 E30 strains sequenced in our study belonged to 2 distinct genogroups, V and VIIb, and showed close relationship with E30 strains circulating in Russia and elsewhere in Asia, including China (Figure 4).

In additional to enterovirus sequences, a gemycircularvirus genome was obtained from a 12-year-old boy. Phylogenetic analysis revealed that this gemycircularvirus strain was closely related to a gemycircularvirus species previously found in CSF sample from a patient with a CNS infection of unknown origin in Sri Lanka (*12*); the level of amino acid identities between the 2 strains were 98.79% for replication-coding sequences and 99.3% for capsid protein–coding sequences.

## Discussion

We describe a viral mNGS investigation characterizing the human virome in CSF of 204 patients in Vietnam with suspected CNS infection of unknown origin. We successfully detected 4 human viral pathogens (enteroviruses, HIV, HBV, and MCV) and 1 virus species (gemycircularvirus) of unknown tropism and pathogenicity in a total of 30 (14.7%) patients. Most patients therefore remained without a known etiology, underscoring the ongoing challenge in identifying a plausible viral pathogen in CSF of patients with CNS infections.

Enteroviruses were the most common viruses, found in 11.3% (23/204) of all analyzed patients (Figure 2), most of whom were children and young adults. This age distribution of enterovirus-infected patients is consistent with observational data from a previous report from Vietnam (*6*), although the median age was slightly higher compared with data from other countries (*13*,*14*). Geographically, all the enterovirus-infected patients were admitted to hospitals from central and highland Vietnam, and none was from southern Vietnam. The underlying mechanism determining this observed spatial pattern of enterovirus-positive cases in this study remains unknown. Our sampling timescale perhaps was not long enough to capture the circulation of enteroviruses in Dong Thap Province. Enteroviruses were previously reported as a leading cause of CNS infection across central and southern Vietnam (*6*,*15*,*16*). Collectively, our findings suggest that reverse transcription PCR (RT-PCR) testing for enteroviruses should be considered in children and young adults with CNS infections.

Of the detected enteroviruses, E30 was the most common serotype. E30 is a well-known pathogen of pediatric aseptic meningitis worldwide (*17*). Phylogenetically, at global scale, E30 belongs to 2 different lineages with distinct patterns of circulation and spread, 1 with a global distribution and the other with geographic restriction within Asia (*17*). The cocirculation of 2 E30 lineages in Vietnam suggests that E30 was imported into Vietnam on at least 2 occasions. Our analyses thus also contribute to the body of knowledge about the genetic diversity of E30 strains circulating in Vietnam.

The detection of bloodborne viruses such as HBV and HIV is unlikely to have a direct link with patients’ neurologic symptoms, although HBV has previously been reported in CSF of patients with CNS infections of unknown origin (*18*). The detection of HIV in CSF might have been a consequence of traumatic tap occurring during the lumbar puncture, as reflected by the high number of red blood cells in 1 of 2 HIV-positive CSF samples (data not shown). However, neuroinvasion of HIV has also been reported (*19*). Likewise, the pathogenic potential of a gemycircularvirus genome requires further investigation, although the detection of the gemycircularvirus genome in CSF has been reported in several papers (*12*,*18*,*20*). The detection of MCV and papillomavirus in CSF might result from contamination of viral skin flora during lumbar puncture.

Similar to previous reports about discrepancy between mNGS and conventional diagnostic testing (*8*,*18*,*21*), our observations found that most mNGS-positive results were not confirmed by subsequent viral RT-PCR assays, especially the sensitive enterovirus-specific RT-PCR with a limit of detection of ≈9 copies/reaction (*22*). Such results could be attributable to bleedover (also called index hopping) of indices from reads of 1 sample into reads of another sample co-sequenced on the same Illumina run (R. Sinha et al., unpub. data, https://doi.org/10.1101/125724). Applying double indexes, which was not used in our study, has been shown to substantially reduce, but not eliminate, the cross-contamination phenomenon between samples in the same run.

Our study has some limitations. First, as outlined previously, we did not employ a double unique index combination strategy per sample as part of the sequencing procedure. The well-known index hopping phenomenon possibly explains the high discrepancy between confirmatory PCR and mNGS results (*21*,*23*,*24*) and emphasizes the usefulness of dual indexing and including no template controls. As such, we pragmatically chose to verify our mNGS by performing specific PCR on original materials. Second, the DNase treatment step in our assay meant to reduce cellular DNA concentration in CSF might reduce the sensitivity of mNGS for the detection of DNA viruses such as herpes simplex virus (*25*,*26*). Third, some of the non–PCR-confirmed viral sequences likely originated from contamination of reagents, which is a lingering problem for mNGS (*27*,*28*).

In summary, our results emphasize that mNGS could detect a broad range of viral nucleic acids in CSF. In spite of extensive investigation, establishing the etiology in many patients with CNS infections remains a challenge. However, our findings indicate that enteroviruses are important causes of viral CNS infections in Vietnam and thus should be considered in the differential diagnosis among young patients with CNS infections.

Appendix Additional information about viral metagenomic analysis of cerebrospinal fluid from patients with acute central nervous system infections of unknown origin, Vietnam.
