# Supplementary material for: Viral Metagenomic Analysis of Cerebrospinal Fluid from Patients with Acute Central Nervous System Infections of Unknown Origin, Vietnam
Source: Emerg Infect Dis. 2021 Jan;27(1):205–13. doi: 10.3201/eid2701.202723 (PMC7774551; doi:10.3201/eid2701.202723)
Supplement: Appendix — Additional information about viral metagenomic analysis of cerebrospinal fluid from patients with acute central nervous system infections of unknown origin, Vietnam. [file 20-2723-Techapp-s1.pdf]

Article DOI: https://doi.org/10.3201/eid2701.202723

Viral Metagenomic Analysis of Cerebrospinal Fluid from Patients with Acute Central Nervous System Infections of Unknown Origin, Vietnam

Appendix

Appendix Table 1. List of diagnostic assays conducted by the original study

Pathogens*	Assay	References
Dengue virus	ELISA	(*1*)
Japanese encephalitis virus	ELISA	(*1*)
Herpes simplex virus 1 and 2	PCR	(*2*)
Varicella-zoster virus	PCR	(*3*)
Enterovirus	PCR	(*4*)
Neisseria meningitidis	PCR	(*5*)
Streptococcus pneumoniae	PCR	(*5*)
Hemophilia influenza type b	PCR	(*5*)
Streptococcus suis	PCR	(*6*)

*Diagnostic tests performed in every case as part of standard of care at participating hospitals including complete blood count, blood culture, gram/ZN smears, CSF culture and CSF examination if patients have neurologic symptoms and CNS infection is suspected.

Appendix Table 2. List of primers and probes used for PCR confirmatory

Viruses	Oligo sequence (5′→3′)			Sources
	Forward	Reverse	Probe	
HBV	GGACCCCTGCTCGTGTTACA	GAGAGAAGTCCACCMCGAGTCTAGA	FAM-TGTTGACAARAATCCTCACAATACCRCAGA-TAMRA	Newly designed
Rotavirus	ACC ATC TWC ACR TRA CCC TC	GGT CAC ATA ACG CCC CTA TA	FAM-ATGAGCACAATAGTTAAAAGCTAACACTGT CAA-BHQ1	(*7*)
Enterovirus	CCCTGAATGCGGCTAAT	ATTGTCACCATAAGCAGCC	CY5-ACCCAAAGTAGTCGGTTCCG -BHQ3	(*8*)
HIV1	GGTGCGAGAGCGTC	ATGCTRTCATCATYTCTTC		(*9*)
	ATGGGTRAARGTARTAGAAGAAAAGGG	CTGCCTGRTGYCCYCCCACTA		
Gemycircularvirus	GTGGTAATGGTCGTCGGTATTC	CCTCATCATTCGTAGTAAGCAATCTCA		(*10*)
	AGTCCTGAATGTTTCCACTCG	CAAGCGTTCCCTCGAAAATGAC		Newly designed
Cyclovirus VN	GAGCGCACATTGAAAGAGCTAAA	TCTCCTCCTTCAATGACAGAAACAAC	FAM-CGADAATAAGGMATACTGCTCTAAAGSTGGCG-BHQ1	(*11*)
Molluscum contagiosum virus	AACCTACGCTACCTGAAGMTGGA	CAGGCTCTTGATGGTCGARATGGA		(*12*)

Appendix Table 3. Baseline characteristics, and CSF white cell count of patients with viruses detected by mNGS

Characteristic	Hepatitis B virus (n = 3)	Human immunodeficiency virus (n = 2)	Molluscum contagiosum virus (n = 1)	Gemycircularvirus (n = 1)
Male, no. (%)	3 (100)	2 (100)	1 (100)	1 (100)
Age (median; min-max)	42 (16–63)	57.5 (49–66)	7	12
Location				
Hue, no. (%)	0	0	0	0
Dak lak, no. (%)	0	0	1 (100)	0
Khanh Hoa, no. (%)	2 (75)	0	0	0
Dong Thap, no. (%)	1 (25)	2 (100)	0	1 (100)
3-d fever (at enrollment or last 3 days)				
Fever, no. (%); median (min-max)	3 (100); 38 (38–39)	1 (50); 39	1 (100); 38.5	0
Fever with unknown temp, no. (%)	0	0	0	1 (100)
No fever, no. (%)	0	1 (50)	0	0
Unknown, no. (%)	0	0	0	0
Outcome				
Death or discharged to die, no. (%)	0	0		
Discharge with complete recovery, no. (%)	2 (75)	0	1 (100)	1 (100)
Discharged with incomplete recovery, no. (%)	1 (25)	1 (50)	0	0
Transferred to another hospital, no. (%)	0	1 (50)	0	0
Other (patient request), no. (%)	0	0	0	0
Unknown, no. (%)	0	0	0	0
CSF White Cells (cell/mm^3) Median (min-max)	1530 (7–2590)	835 (340–1330)	70	60

References

1. Cardosa MJ, Wang SM, Sum MSH, Tio PH. Antibodies against prM protein distinguish between previous infection with dengue and Japanese encephalitis viruses. BMC Microbiol. 2002;2:9. PubMed https://doi.org/10.1186/1471-2180-2-9

2. van Doornum GJJ, Guldemeester J, Osterhaus ADME, Niesters HGM. Diagnosing herpesvirus infections by real-time amplification and rapid culture. J Clin Microbiol. 2003;41:576–80. PubMed https://doi.org/10.1128/JCM.41.2.576-580.2003

3. de Jong MD, Weel JF, Schuurman T, Wertheim-van Dillen PM, Boom R. Quantitation of varicella-zoster virus DNA in whole blood, plasma, and serum by PCR and electrochemiluminescence. J Clin Microbiol. 2000;38:2568–73. PubMed https://doi.org/10.1128/JCM.38.7.2568-2573.2000

4. Beld M, Minnaar R, Weel J, Sol C, Damen M, van der Avoort H, et al. Highly sensitive assay for detection of enterovirus in clinical specimens by reverse transcription-PCR with an armored RNA internal control. J Clin Microbiol. 2004;42:3059–64. PubMed https://doi.org/10.1128/JCM.42.7.3059-3064.2004

5. Corless CE, Guiver M, Borrow R, Edwards-Jones V, Fox AJ, Kaczmarski EB. Simultaneous detection of Neisseria meningitidis, Haemophilus influenzae, and Streptococcus pneumoniae in suspected cases of meningitis and septicemia using real-time PCR. J Clin Microbiol. 2001;39:1553–8. PubMed https://doi.org/10.1128/JCM.39.4.1553-1558.2001

6. Nga TVT, Nghia HDT, Tu TP, Diep TS, Mai NTH, Chau TTH, et al. Real-time PCR for detection of Streptococcus suis serotype 2 in cerebrospinal fluid of human patients with meningitis. Diagn Microbiol Infect Dis. 2011;70:461–7. PubMed https://doi.org/10.1016/j.diagmicrobio.2010.12.015

7. Dung TTN, Phat VV, Nga TVT, My PVT, Duy PT, Campbell JI, et al. The validation and utility of a quantitative one-step multiplex RT real-time PCR targeting rotavirus A and norovirus. J Virol Methods. 2013;187:138–43. PubMed https://doi.org/10.1016/j.jviromet.2012.09.021

8. Thanh TT, Anh NT, Tham NT, Van HM, Sabanathan S, Qui PT, et al. Validation and utilization of an internally controlled multiplex Real-time RT-PCR assay for simultaneous detection of enteroviruses and enterovirus A71 associated with hand foot and mouth disease. Virol J. 2015;12:85. PubMed https://doi.org/10.1186/s12985-015-0316-2

9. Chook JB, Ong LY, Takebe Y, Chan KG, Choo M, Kamarulzaman A, et al. Molecular detection of HIV-1 subtype B, CRF01_AE, CRF33_01B, and newly emerging recombinant lineages in Malaysia. Am J Trop Med Hyg. 2015;92:507–12. PubMed https://doi.org/10.4269/ajtmh.14-0681

10. Phan TG, Mori D, Deng X, Rajidrajith S, Ranawaka U, Fan Ng TF, et al. Small viral genomes in unexplained cases of human encephalitis, diarrhea, and in untreated sewage. Virology. 2015;482:98–104. PubMed https://doi.org/10.1016/j.virol.2015.03.011

11. Van Tan L, Van Doorn HR, Trung D, Hong T, Phuong T, De Vries M. Identification of a New Cyclovirus in Cerebrospinal Fluid of Patients with Acute Central Nervus System Infections. MBio. 2013;4:1–10.

12. Hošnjak L, Kocjan BJ, Kušar B, Seme K, Poljak M. Rapid detection and typing of Molluscum contagiosum virus by FRET-based real-time PCR. J Virol Methods. 2013;187:431–4. PubMed https://doi.org/10.1016/j.jviromet.2012.11.008
